# A Bayesian Adaptive Design in Cancer Phase I Trials Using Dose Combinations with Ordinal Toxicity Grades

**DOI:** 10.3390/stats3030017

**Published:** 2020-07-17

**Authors:** Márcio A Diniz, Sungjin Kim, Mourad Tighiouart

**Affiliations:** Biostatistics and Bioinformatics Research Center, Samuel Oschin Compreenhensive Cancer Institute, Cedars-Sinai Medical Center, 700 N. San Vicente Blvd, Los Angeles, CA 90069, USA

**Keywords:** phase I designs, drug combination, ordinal toxicity, Escalation With Overdose Control, Continual Reassessment Method

## Abstract

We propose a Bayesian adaptive design for early phase drug combination cancer trials incorporating ordinal grade of toxicities. Parametric models are used to describe the relationship between the dose combinations and the probabilities of the ordinal toxicities under the proportional odds assumption. Trial design proceeds by treating cohorts of two patients simultaneously receiving different dose combinations. Specifically, at each stage of the trial, we seek the dose of one agent by minimizing the Bayes risk with respect to a loss function given the current dose of the other agent. We consider two types of loss functions corresponding to the Continual Reassessment Method (CRM) and Escalation with Overdose Control (EWOC). At the end of the trial, we estimate the MTD curve as a function of Bayes estimates of the model parameters. We evaluate design operating characteristics in terms of safety of the trial and percent of dose recommendation at dose combination neighborhoods around the true MTD by comparing this design to the one that uses a binary indicator of DLT. The methodology is further adapted to the case of a pre-specified discrete set of dose combinations.

## 1. Introduction

The primary goal of early phase cancer clinical trials, also known as phase I trials, is to estimate the maximum tolerated dose (MTD) of a new drug or combination of drugs for use in larger randomized phase II/III trials. Dose escalation is guided using dose limiting toxicity (DLT) outcomes from all previously treated patients. The definition of DLT is pre-specified in the clinical protocol and consists of serious adverse events usually classified as Grade 3 or higher in the Common Toxicity Criteria for Adverse Events (CTCAE) [1]. The CTCAE is a systematic classification system proposed by the National Cancer Institute to guide investigators in identifying and evaluate the severity of adverse events varying from mild (Grade 1) to death (Grade 5). Even though such criteria have been extensively adopted allowing investigators to better understand the toxicity profiles of patients, classical cancer phase I designs dichotomize patients’ toxicity profiles based on the maximum grade of DLT as 0–2 (absence) and 3–5 (presence). Dichotomization is convenient for statistical modeling, but also entails loss of information and it should be avoided.

For single agent dose finding trials in cancer, many authors have investigated properties of statistical models and designs that account for all toxicity grades experienced by patients in the trial. Some of these use multivariable models for eliciting the different grades of toxicities as a function of dose [2–8] and others proposed summary indexes to account for different types of toxicities using weights defined by clinicians [9–16]. In general, there is a modest gain in safety and efficiency of the trial under some scenarios. In the first approach, we highlight the work of Van Meter et al. [6] that extended the Continual Reassessment Method (CRM) under the assumption of proportional odds considering toxicities Grades 0, 1, 2, 3 and 4–5, and Tighiouart et al. [8] that proposed the proportional odds Escalation With Overdose Control (EWOC) modeling toxicities 0–1, 2 and 3–5. They both showed some benefits either in safety or precision of the MTD estimate when compared to the classical designs [17–21] for single agent trials under certain scenarios.

Even though dose-finding designs for two agents have been the focus of statistical research in the last two decades [22–34], the proposed approaches have ignored lower grades and different types of toxicities. Noteworthy, Tighiouart et al. [34] presented an early phase I EWOC design that estimates an MTD curve lying anywhere within the Cartesian plane defined by the range of the continuous doses of two synergistic agents, and Diniz et al. [35] investigated properties of this approach using the CRM criterion. In this paper, we extend the work of Tighiouart et al. [8] by accounting for lower grades of toxicities in the designs described in [34,35]. We assess the benefits of this added level of model complexity by comparing safety of the trial and efficiency of the estimate of the MTD to the ones obtained using binary DLT. We note that Tighiouart et al. [8] showed a desirable ethical property that controls the magnitude of the escalation for the continuous dose level in the absence of DLT. More precisely, they showed that the escalation is lower for a patient who exhibits a Grade 2 DLT than the size of this dose level had this patient experienced a 0–1 grade DLT. This property does not hold in the current setting partly due to the overlapping nature of DLTs in cancer treatment with drug combinations. Nevertheless, we show that a similar characteristic of including lower grades of toxicities result in a more cautious dose escalation when the true MTD is far from the minimum dose combination without loss of efficiency, and hence results in lower DLTs relative to the binary DLT model.

The manuscript is organized as follows. Section 2 describes the proportional odds model for two drugs and trial designs using EWOC and CRM schemes. We present the simulation scenarios and design operating characteristics for the ordinal and binary toxicity models in Section 3. We illustrate how the method is adapted to a set of discrete dose levels in Section 4 and conclude with a discussion and final recommendations in Section 5.

## 2. Method

### 2.1. Dose-Toxicity Model

Let *G* = 0, 1,...,4 be the maximum toxicity grade experienced by a patient during one cycle of therapy, and define DLT as a maximum of Grade 3 or 4 toxicity. Let *Z* be the aggregated maximum grade of toxicity defined by

(1) $$Z=\{\begin{array}{ll} 0 & \text{if}\;G=0,1\\ 1 & \text{if}\;G=2\\ 2 & \text{if}\;G=3,4. \end{array}$$

Cytotoxic agents are denoted by *A* with doses *x* ∈ [*X*_*min*_, *X*_*max*_] and *B* with doses *y* ∈ [*Y*_*min*_, *Y*_*max*_]. We consider the family of dose-toxicity models

(2) $$P(Z\geq z|x,y)=F\left(\alpha_{z}+\beta x+\gamma y+\eta xy\right)\;\text{for}\;z=1,2,$$

where *F*(.) is a known cumulative distribution function (c.d.f.); *α*_1_ is the probability of *G* ≥ 2 and *α*_2_ is the probability of *G* ≥ 3, 4 at the minimum dose combination; *β*, *γ* are the effects of drugs *A* and *B*, respectively; and *η* quantifies the extent of synergy between the two drugs. The doses *x* and *y* are standardized to be in the interval [0, 1] so that (0, 0) corresponds to the minimum dose combination available in the trial (*X*_*min*_, *Y*_*min*_). We assume that the probability of DLT increases with the dose of any one of the agents when the other one is held constant. A necessary and sufficient condition for this to hold is to assume that *β* > 0, *γ* > 0, and *η* > 0. In addition, *α*_2_ ≤ *α*_1_ since *F* is non-decreasing. The MTD is defined as any dose combination (x*, y*) that satisfies

(3) $$P\left(Z=2|x^{*},y^{*}\right)=\theta ,$$

where *θ* is the target probability of DLT and is pre-specified by the clinicians. This target depends on the severity and clinical manageability of DLT; it is usually set relatively high when the DLT is a transient, correctable or nonfatal condition and low when it is fatal or life threatening.

Then, a set *C* of dose combinations can be characterized as MTD from (2) and (3),

(4) $$C=\left\{\left(x^{*},y^{*}\right):y^{*}=\frac{F^{-1}(\theta )-\alpha_{2}-\beta x^{*}}{\gamma +\eta x^{*}}\right\}.$$

We further reparameterize Model (2) in terms of parameters that are easily understood by clinicians: *ρ*_200_ is the probability of Grade 3 or 4 toxicity (DLT) at the minimum dose combination (0, 0), *ρ*_100_ is the probability of Grade 2 or more toxicity at dose (0, 0), *ρ*_210_ is the probability of Grade 3 or 4 toxicity (DLT) at dose (1, 0) and *ρ*_201_ is the probability of Grade 3 or 4 toxicity (DLT) at dose (0, 1). Other reparametrizations are also possible. The restrictions *β*, *γ* > 0 and *α*_2_ ≤ *α*_1_ translate into *ρ*_200_ < *min*{*ρ*_210_, *ρ*_201_} and *ρ*_200_ ≤ *ρ*_100_, respectively. It then follows that

(5) $$\alpha_{1}=F^{-1}\left(\rho_{100}\right)\;\alpha_{2}=F^{-1}\left(\rho_{200}\right)\;\beta =F^{-1}\left(\rho_{210}\right)-F^{-1}\left(\rho_{200}\right)\;\gamma =F^{-1}\left(\rho_{201}\right)-F^{-1}\left(\rho_{200}\right).$$

Similarly, the MTD set can be rewritten as

(6) $$C=\left\{\left(x^{*},y^{*}\right):y^{*}=\frac{F^{-1}(\theta )-F^{-1}\left(\rho_{200}\right)-\left[F^{-1}\left(\rho_{210}\right)-F^{-1}\left(\rho_{200}\right)\right]x^{*}}{\left[F^{-1}\left(\rho_{201}\right)-F^{-1}\left(\rho_{200}\right)\right]+\eta x^{*}}\right\}.$$

### 2.2. Prior and Posterior Distributions

To easily elicit prior information from single agent phase I trials, we assume that *ρ*_100_, *ρ*_210_, *ρ*_201_ are independent a priori with *ρ*_100_ ~ *Beta*(*a*_100_, *b*_100_), *ρ*_210_ ~ *Beta*(*a*_210_, *b*_210_), *ρ*_201_ ~ *Beta*(*a*_201_, *b*_201_), and given {*ρ*_210_, *ρ*_201_, *ρ*_100_}, *ρ*_200_/*min*{*ρ*_210_, *ρ*_201_, *ρ*_100_} ~ *Beta*(*a*_200_, *b*_200_). The prior distribution for the interaction parameter *η* is given by a *Gamma* distribution with mean *E*(*η*) = *a*/*b* and variance *Var*(*η*) = *a*/*b*^2^.

Let $D_{n}=\left\{\left(x_{i},y_{i},z_{i}\right),i=1,\ldots ,n\right\}$ be the data after enrolling *n* patients in the trial. Using Bayes rule, the posterior distribution of the model parameters is proportional to the product of the likelihood and prior distribution

(7) $$\pi \left(\rho_{210},\rho_{201},\rho_{200},\rho_{100},\eta |D_{n}\right)\;\;\propto \prod_{i=1}^{n}\{H_{1}{\left(\rho_{210},\rho_{201},\rho_{200},\rho_{100},\eta ;x_{i},y_{i}\right)}^{I\left(Z_{i}=0\right)}\;\;\times {\left[H_{1}\left(\rho_{210},\rho_{201},\rho_{200},\rho_{100},\eta ;x_{i},y_{i}\right)-H_{2}\left(\rho_{210},\rho_{201},\rho_{200},\eta ;x_{i},y_{i}\right)\right]}^{I\left(Z_{i}=1\right)}\;\;\times H_{2}{\left(\rho_{210},\rho_{201},\rho_{200},\eta ;x_{i},y_{i}\right)}^{I\left(Z_{i}=2\right)}\}\pi \left(\rho_{210},\rho_{201},\rho_{200},\rho_{100},\eta \right),$$

where

$$H_{1}\left(\rho_{210},\rho_{201},\rho_{200},\rho_{100},\eta ;x,y\right)\;\;=1-F\left(F^{-1}\left(\rho_{100}\right)+\left[F^{-1}\left(\rho_{210}\right)-F^{-1}\left(\rho_{200}\right)\right]x+\left[F^{-1}\left(\rho_{201}\right)-F^{-1}\left(\rho_{200}\right)\right]y+\eta xy\right),\;H_{2}\left(\rho_{210},\rho_{201},\rho_{200},\eta ;x,y\right)\;\;=1-F\left(F^{-1}\left(\rho_{200}\right)+\left[F^{-1}\left(\rho_{210}\right)-F^{-1}\left(\rho_{200}\right)\right]x+\left[F^{-1}\left(\rho_{201}\right)-F^{-1}\left(\rho_{200}\right)\right]y+\eta xy\right).$$

We used JAGS [36] to sample from the posterior distribution of these parameters and estimate design operating characteristics of the designs described below.

### 2.3. Trial Design

The dose allocation algorithm proceeds by treating cohorts of two patients simultaneously. The dose combinations assigned to newly enrolled patients are based on EWOC scheme and the CRM principle proposed by the authors of [34,35], respectively.

1. Each patient in the first cohort of two patients receives the same dose combination (*x*_1_, *y*_1_) = (*x*_2_, *y*_2_) = (0, 0).
2. In the *i*th cohort of two patients:
  (a) If *i* is even, then patient 2*i* − 1 receives dose (*x*_2*i*−1_, *y*_2*i*−3_) and patient 2*i* receives dose (*x*_2*i*−2_, *y*_2*i*_), where
    $$x_{2i-1}=\pi_{\Gamma_{A|B=y_{2i-3}}}^{-1}\left(\alpha |D_{i-1}\right)\;\;y_{2i}=\pi_{\Gamma_{B|A=x_{2i-2}}}^{-1}\left(\alpha |D_{i-1}\right)$$
    for EWOC criterion.
    $$x_{2i-1}=\underset{x}{\text{argmin}}\left|H\left({\hat{\rho }}_{200},{\hat{\rho }}_{201},{\hat{\rho }}_{210},{\hat{\rho }}_{100},\hat{\eta };x,y_{2i-3}\right)-\theta \right|\;\;y_{2i}=\underset{y}{\text{argmin}}\left|H\left({\hat{\rho }}_{200},{\hat{\rho }}_{201},{\hat{\rho }}_{210},{\hat{\rho }}_{100},\hat{\eta };x_{2i-2},y\right)-\theta \right|$$
    for CRM principle.
  (b) If *i* is odd, then patient 2*i* − 1 receives dose (*x*_2*i*−3_, *y*_2*i*−1_) and patient 2*i* receives dose (*x*_2*i*_, *y*_2*i*−2_), where
    $$\;x_{2i}=\pi_{\Gamma_{A|B=y_{2i-2}}}^{-1}\left(\alpha |D_{i-1}\right)\;y_{2i-1}=\pi_{\Gamma_{B|A=y_{2i-3}}}^{-1}\left(\alpha |D_{i-1}\right)$$
    for EWOC criterion.
    $$\;x_{2i}=\underset{r}{\text{argmin}}\left|H\left({\hat{\rho }}_{200},{\hat{\rho }}_{201},{\hat{\rho }}_{210},{\hat{\rho }}_{100},\hat{\eta };x,y_{2i}\right)-\theta \right|\;y_{2i-1}=\underset{y}{\text{argmin}}\left|H\left({\hat{\rho }}_{200},{\hat{\rho }}_{201},{\hat{\rho }}_{210},{\hat{\rho }}_{100},\hat{\eta };x_{2i-1},y\right)-\theta \right|$$
    for CRM principle.
3. Repeat Step 2 until *n* patients are enrolled to the trial subject to the following stopping rule.

Here, $\pi_{\Gamma_{A|B=y}}^{-1}(\cdot |D)$ denotes the inverse c.d.f of the posterior distribution of the MTD of drug *A* given the level of drug *B* = *y* and ${\hat{\rho }}_{q},\hat{\eta },q\in \{200,201,210,100\}$ are the posterior medians.

#### Stopping rule:

We stop enrollment to the trial if $\text{P}\left(P(\text{DLT}|(x,y)=(0,0))>\theta +\delta_{1}|\text{data}\right)>\delta_{2},$ i.e. if the posterior probability that the probability of DLT at the minimum available dose combination in the trial exceeds the target probability of DLT is high. The parameters *δ*_1_ and *δ*_2_ are design parameters chosen to achieve desirable model operating characteristics.

At the end of the trial, we estimate the MTD curve using Bayes estimates of the parameters defining this curve as

(8) $$\hat{C}=\left\{\left(x^{*},y^{*}\right):y^{*}=\frac{F^{-1}(\theta )-F^{-1}\left({\hat{\rho }}_{200}\right)-\left[F^{-1}\left({\hat{\rho }}_{210}\right)-F^{-1}\left({\hat{\rho }}_{200}\right)\right]x^{*}}{\left[F^{-1}\left({\hat{\rho }}_{201}\right)-F^{-1}\left({\hat{\rho }}_{200}\right)\right]+\eta x^{*}}\right\},$$

where ${\hat{\rho }}_{200},{\hat{\rho }}_{100},{\hat{\rho }}_{210},{\hat{\rho }}_{201},\hat{\eta }$ are the posterior medians given the data *D*_*n*_.

When using EWOC criteria, we seek a dose such that the posterior probability that the MTD exceeds this dose is bounded by a feasibility bound *α*. For example, when *i* is even, the dose of drug *A*, *x*^★^, assigned to patient (2*i* − 1) is the maximum dose level of *A* such that the posterior probability that the MTD of *A* given that the level of drug *B* is *y*_2*i*−3_ exceeds *x*^★^ is bounded by *α*, i.e., $x^{⋆}=x_{2i-1}=\pi_{\Gamma_{A|B=y_{2i-3}}}^{-1}\left(\alpha |D_{i-1}\right).$ On the other hand, CRM principle consists of estimating the model parameters by the median of the posterior distribution, and then assigning the dose *x*^★^ that minimizes the distance between the estimated probability of DLT and the target risk of DLT *θ*, $\left|H\left({\hat{\rho }}_{200},{\hat{\rho }}_{201},{\hat{\rho }}_{210},{\hat{\rho }}_{100},\hat{\eta };x^{⋆},y_{2i-3}\right)-\theta \right|.$

## 3. Simulations

### 3.1. Set-Up and Scenarios

**Figure 1.**
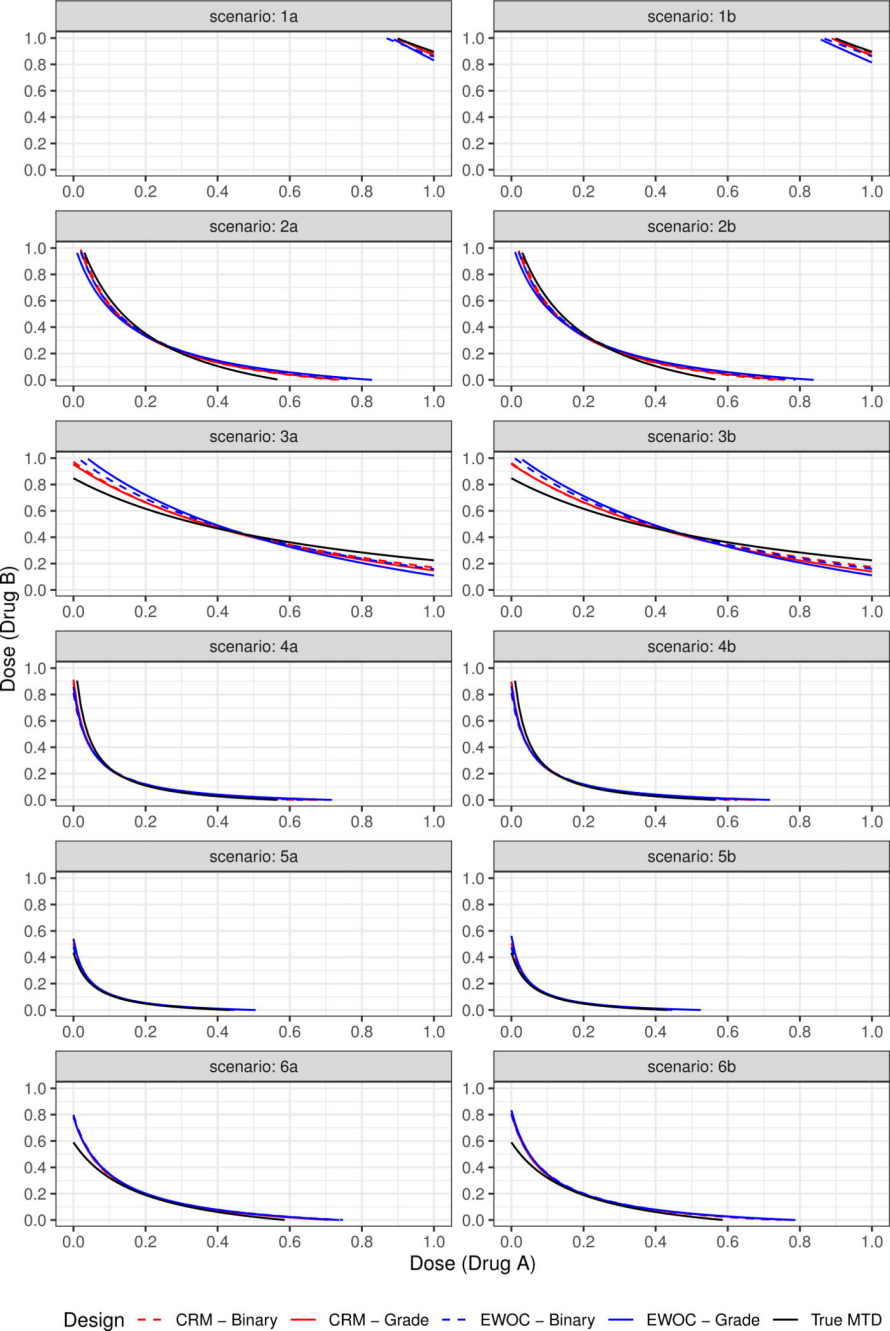
True and estimated MTD curves under Scenarios (1)–(6).

We study the performance of these designs in six pairs of scenarios as determined by the true parameter values (*ρ*_100_, *ρ*_200_, *ρ*_210_, *ρ*_201_, *η*). In all cases, the target probability of DLT is fixed at *θ* = 0.33 and the trial sample size is *n* = 42 patients. The feasibility bound *α* is set to 0.25 at the start of the trial and increases in increments of 0.05 each time a cohort of two patients are enrolled to a maximum value of 0.5. We investigate the influence of the percentage of Grade 2, defined as *P*(*Z* = 2) = *ρ*_100_ − *ρ*_200_, considering two possible values for *ρ*_100_ = 0.5, 0.9 for each pair of scenarios. Hence, each pair will have the same true MTD curve (see Figure 1). Scenario (1) (*ρ*_100_, 10^−7^, 3 × 10^−6^, 3 × 10^−6^, 10) shows two drugs that are very safe within the range of available doses in the trial where the true MTD curve lies near the upper-right corner of the x–y plane. In Scenario (2) (*ρ*_100_, 0.01, 0.9, 0.2, 20), the MTD of Agent A when Agent B is at its minimum dose level is within the range of doses of Agent A, but the MTD of Agent B when Agent A is at its minimum dose level is above the maximum dose level of Agent B. For Scenario (3) (*ρ*_100_, 0.001, 0.01, 0.6, 20), Drug A is very safe, but the MTD of Agent B when Drug A is at its minimum dose level is just above 0.8. Scenario (4) (*ρ*_100_, 0.01, 0.9, 0.2, 100) is similar to Scenario (2) except that the two drugs are highly synergistic. Scenario (5) (*ρ*_100_, 0.2, 0.9, 0.9, 100) is a case where the middle of true MTD curve is close to the initial dose (0, 0) with high probability of Grade 2 toxicity. Finally, Scenario (6) (*ρ*_100_, 0.2, 0.57, 0.57, 20) is similar to Scenario (5) except that the interaction between the two drugs is much smaller.

### 3.2. Operating Characteristics

We evaluate the performance of the two designs using EWOC and CRM criteria by assessing the safety of the trial designs as well as the efficiency of the estimate of the MTD curve based on 3000 simulated trials.

#### 3.2.1. Safety

We assess trial safety by reporting the average percent of Grade 2 and 3 DLT across all 3000 trials and the percent of trials that have a DLT rate exceeding *θ* + *δ*, for *δ* = 0.1. The threshold *θ* + 0.1 is used as an indicator of an excessive DLT rate.

#### 3.2.2. Efficiency

We present an estimate of the MTD curve using the average posterior medians of the model parameters. Under the reparameterization, the estimate is

(9) $$\hat{C}=\left\{\left(x^{*},y^{*}\right):y^{*}=\frac{\left(F^{-1}(\theta )-F^{-1}\left({\hat{\rho }}_{200}\right)\right)-\left(F^{-1}\left({\hat{\rho }}_{210}\right)-F^{-1}\left({\hat{\rho }}_{200}\right)\right)x^{*}}{\left(F^{-1}\left({\hat{\rho }}_{201}\right)-F^{-1}\left({\hat{\rho }}_{200}\right)\right)+\hat{\eta }x^{*}}\right\},$$

where *F*(.) is the logistic function and ${\hat{\rho }}_{200},{\hat{\rho }}_{201},{\hat{\rho }}_{210},\hat{\eta }$ are the average posterior medians of the parameters *ρ*_200_, *ρ*_201_, *ρ*_210_, *η* from all *m* = 3000 trial replicates.

The MTD curves lie in a two-dimensional plan, therefore closeness between two curves can be measured based on several approaches. We calculate two measures of efficiency introduced by Tighiouart et al. [32,34,37] and applied to real trials in [38,39]. The first one is the pointwise average relative minimum distance from the true MTD curve to the estimated MTD curve. Let *C*_*i*_ be the estimated MTD curve and *C*_*true*_ be the true MTD curve for *i* = 1,...,*m*. For every point (*x*, *y*) ∈ *C*_*true*_, let

(10) $$d_{\left(x,y\right)}^{\left(i\right)}=\mathrm{sign}\left(y^{\prime }-y\right)\times \min_{\left\{\left(x^{*},y^{*}\right):\left(x^{*},y^{*}\right)\in C_{i}\right\}}{\left({\left(x-x^{*}\right)}^{2}+{\left(y-y^{*}\right)}^{2}\right)}^{1/2},$$

where *y′* is such that (*x*, *y′*) ∈ *C*_*i*_. This is the minimum relative distance of the point (*x*, *y*) on the true MTD curve to the estimated MTD curve *C*_*i*_. If the point (*x*, *y*) is below *C*_*i*_, then $d_{\left(x,y\right)}^{\left(i\right)}$ is positive. Otherwise, it is negative. Let

(11) $$d_{\left(x,y\right)}=\frac{1}{m}\sum_{i=1}^{m}d_{\left(x,y\right)}^{\left(i\right)}.$$

The distance (11) is the pointwise average relative minimum distance from the true MTD curve to the estimated MTD curve and can be interpreted as the pointwise average bias in estimating the MTD.

As the magnitude of bias is relative to the true MTD value, we also quantify the percentage of trials for which the minimum distance of the point (*x*, *y*) from the true MTD curve to the estimated MTD curve *C*_*i*_ is no more than (100 × *p*)% of the true MTD,

(12) $$P_{\left(x,y\right)}=\frac{1}{m}\sum_{i=1}^{m}I\left(\left|d_{\left(x,y\right)}^{\left(i\right)}\right|\leq p\Delta (x,y)\right),$$

where Δ(*x*, *y*) is the Euclidian distance between the minimum dose combination (0, 0) and the point (*x*, *y*) on the true MTD curve and 0 < *p* < 1.

The geometric idea is to draw a circle with center (*x*, *y*) on the true MTD curve and radius *p*Δ(*x*, *y*), and then the percent of trials with the MTD curve estimate *C*_*i*_ within this circle is given by *P*(*x*, *y*). Therefore, the statistic (12) measures the percentage of trials satisfying this condition for a given 100*p*% tolerance.

### 3.3. Results

Summary statistics for evaluating trial safety are presented in Table 1. In Scenarios (2)–(6), the average percent of DLTs are similar between the binary model and ordinal model using both EWOC and CRM criteria. Under Scenario (1), the binary model results in a higher average percentage of DLTs when compared with the ordinal model for both criteria. A similar trend was observed for single agent dose finding trials by Tighiouart et al. [8] when the true MTD is close to the maximum dose and *ρ*_100_ is high. This can be explained by the fact that when the MTD is very far from the minimum dose, the ordinal dose–toxicity based model design tends to have a more cautious dose escalation towards the MTD relative to the binary model. While this fact was proven for single agent trials by Tighiouart et al. [8], it is not trivial for dose combination trials since these models do not distinguish between DLT attribution to one or both drugs. It may be worth studying the performance of this ordinal model in settings where an unknown fraction of DLTs can be attributed to one or both drugs (see [40]). In all cases, the average percent of DLTs varies between 10.48% and 38.98%, indicating that the trial is safe. This rate is above the target *θ* under Scenario (5) due to the closeness of the MTD curve to the minimum dose combination (0, 0). These findings are also consistent with the percent of trials with an excessive rate of DLTs. This rate is less than 5% in all scenarios except for Scenario (5), where it can reach 13% using the ordinal model and the CRM criteria. We conclude that in general, the trial design is safe except when the true MTD is close to the initial dose.

**Figure 2.**
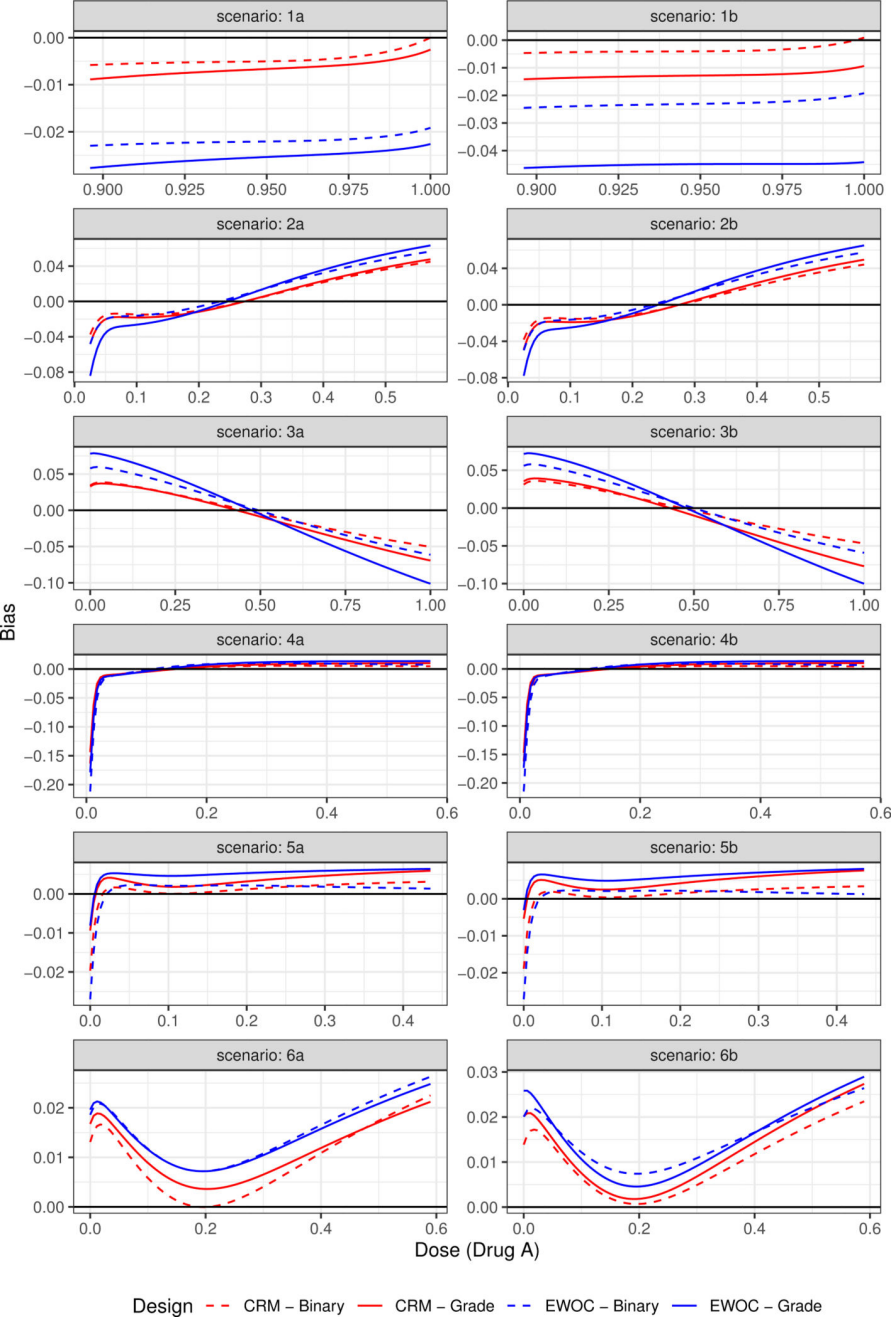
Pointwise average relative minimum distance from the true MTD curve to the estimated MTD curve under Scenarios (1)–(6).

Figure 1 shows the plots of the true and estimated MTD curves obtained using (9). In general, the estimated MTD curves using the binary and ordinal models and EWOC and CRM criteria are close to the true MTD curve, except perhaps near the edges of the true MTD curve. The extent of these differences can be measured by the pointwise average bias shown in Figure 2. Scenarios (2), (3) and (6) show that the pointwise average absolute bias is highest at the edges of the MTD curve and Scenarios (1), (4) and (5) have the highest bias at one extremity of the true MTD curve. In all cases, the extent of differences in pointwise average bias between the binary and ordinal model using both dose estimation criteria are less than 0.04, which is practically not significant as this corresponds to less than 4% of the dose range of either agent.

**Figure 3.**
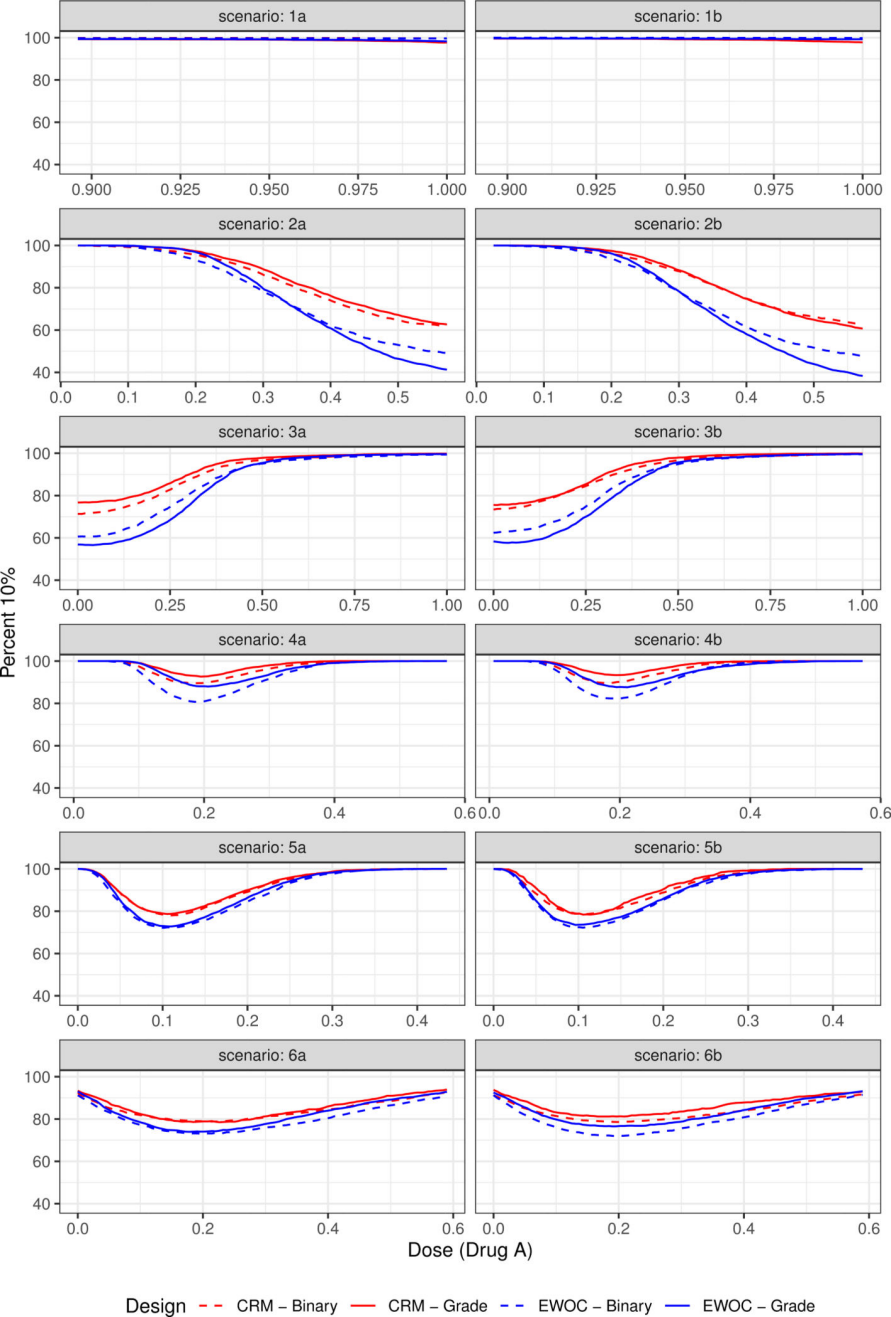
Pointwise percent of MTD recommendation for *p* = 0.1 under Scenarios (1)–(6).

**Figure 4.**
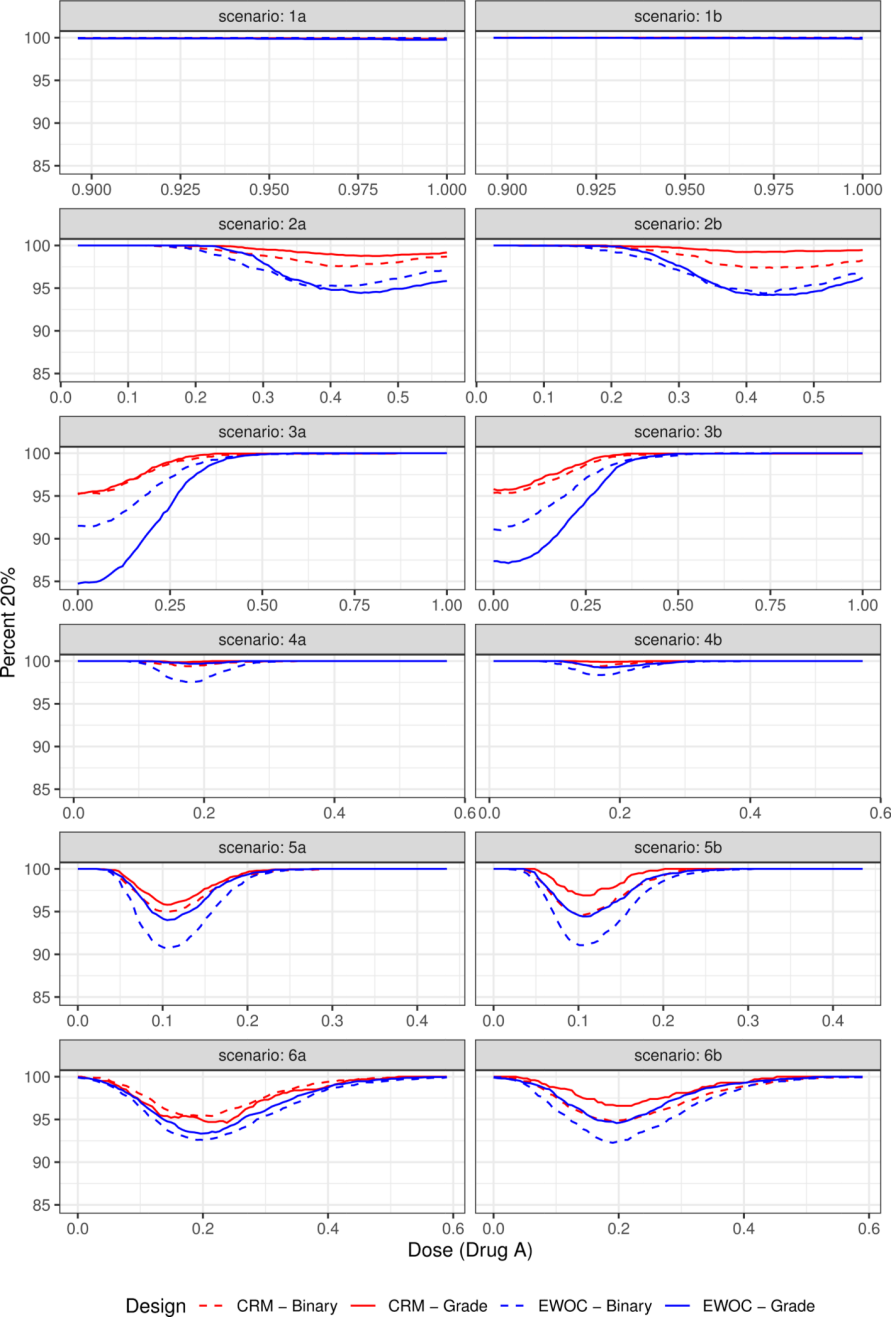
Pointwise percent of MTD recommendation for *p* = 0.2 under Scenarios (1)–(6).

The pointwise percent selection for tolerances *p* = 0.1 and *p* = 0.2 are shown in Figures 3 and 4, respectively. In general, the ordinal and binary models are similar with respect to the pointwise percent selection with the largest differences between 8% and 10% observed under Scenarios (2) and (4) near the edge or middle of the true MTD curve when the tolerance probability is *p* = 0.1. The extent of this difference diminishes with higher tolerance *p* = 0.2, see Figure 4. Moreover, the pointwise percent selection is 85% or more using both models and criteria under all 12 scenarios when *p* = 0.2. We also note that for each EWOC and CRM criteria, the ordinal model has a slightly higher pointwise percent selection relative to the binary model uniformly under Scenarios (4)–(6). Under Scenarios (2) and (3), no model performs uniformly better than the other. Finally, for each ordinal and binary models, CRM outperforms EWOC in the pointwise percent recommendation uniformly across all scenarios with the largest difference of 20% achieved under Scenario (2) using the ordinal model with *p* = 0.1.

The simulation results based on all 12 scenarios favor the use of CRM relative to EWOC to improve the precision of the estimate of the MTD. Given the similarities in the average percent of DLTs and safety of the trial between all models under Scenarios (2)–(6), and the fact that the ordinal model results in much less average percent of DLTs relative to the binary model when the true MTD curve is far away from the initial dose (Scenario (1)) while providing the same level of precision of the estimate of the MTD (Figures 3 and 4), we recommend the use of the ordinal model with CRM criteria for estimating the next dose combinations when designing prospective trials.

## 4. Discrete Approach

For a discrete set of doses, we follow the approach presented by Tighiouart [34]. Let (*x*_1_,…,*x*_*r*_) and (*y*_1_,…,*y*_*s*_) be the doses of Agents *A* and *B*, respectively, with $X_{\min,A}=x_{1},Y_{\min,B}=y_{1}\;\text{and}\;X_{\max,A}=x_{r},Y_{\max,B}=y_{s}$ such that the doses are standardized to be in the interval [0, 1]. Trial design proceeds using the algorithm described in Section 2.3 where the continuous doses recommended in Steps 2 and 3 are rounded to the nearest discrete dose levels. At the end of the trial, a discrete set Γ of dose combinations satisfying (i) and (ii) below is selected as MTDs: Let *C*_*i*_ be the estimated MTD curve at the end of the trial and denote by *d*((*x*_*j*_, *y*_*k*_), *C*_*i*_), the Euclidean distance between the dose combination (*x*_*j*_, *y*_*k*_) and the estimated MTD curve *C*_*i*_.

(i) Let
  $$\Gamma_{A}=\overset{r}{\underset{t=1}{\cup }}\left\{\left(x_{t},y\right):y=\underset{y_{j}}{\mathrm{argmin}}\;d\left(\left(x_{t},y_{j}\right),C_{i}\right)\right\},$$
  $$\Gamma_{B}=\overset{s}{\underset{t=1}{\cup }}\left\{\left(x,y_{t}\right):x=\underset{x_{j}}{\mathrm{argmin}}\;d\left(\left(x_{j},y_{t}\right),C_{i}\right)\right\},\;\text{and}\;\Gamma_{0}=\Gamma_{A}\cap \Gamma_{B}.$$
(ii) Let
  $$\Gamma =\Gamma_{0}\backslash \left\{\left(x^{*},y^{*}\right):P\left(\left|P\left(DLT|\left(x^{*},y^{*}\right)\right)-\theta \right|>\delta_{1}|D_{n}\right)>\delta_{2}\right\}.$$

where $A\backslash B=A\cap B^{C}.$ In (i), dose combinations closest to the MTD are selected by first minimizing the distances across the levels of Drug A, and then across the levels of Drug B. In (ii), we exclude MTDs from (i) that are likely to be either too toxic or too low. The design parameter *δ*_1_ is selected after consultation with a clinician and the parameter *δ*_2_ is selected after exploring a large number of scenarios for a given prospective trial.

### 4.1. Operating Characteristics

The performance of the method is evaluated by calculating the percent of MTDs selection introduced by Tighiouart et al. [34] estimating the percentage that a prospective trial will recommend a set of dose combinations that are all MTDs,

(13) $$\text{PS}=100\times \frac{1}{m}\sum_{i=1}^{m}I\left(\Gamma_{i}\subset \Gamma_{\delta }\right),$$

where $\Gamma_{\delta }=\left\{\left(x_{i},y_{j}\right):\left|P\left(DLT|\left(x_{i},y_{j}\right),z\right)-\theta \right|<\delta \right\}$ is the set of true MTDs such that the threshold parameter *δ* is fixed by a clinician. In the same way, the percentage of selection of at least *K* dose combinations that are MTDs discussed in [35] is

(14) $${\text{PS}}_{K}=100\times \frac{1}{m}\sum_{i=1}^{m}I\left(\left|\Gamma_{i}\cap \Gamma_{\delta }\right|\geq K\right),$$

In addition, the weighted average proportion of the recommended set of dose combinations which are MTDs is given by

(15) $$S_{\Gamma_{\delta }}=\frac{\sum_{i=1}^{m}\left|\Gamma_{i}\cap \Gamma_{\delta }\right|}{\sum_{i=1}^{m}\left|\Gamma_{i}\right|}.$$

The performance of the method is evaluated by calculating the percent of MTDs selection introduced by Tighiouart et al. [34] estimating the percentage that a prospective trial will recommend a set of dose combinations that are all MTDs,

(16) $$\text{PS}=100\times \frac{1}{m}\sum_{i=1}^{m}I\left(\Gamma_{i}\subset \Gamma_{\delta }\right),$$

where $\Gamma_{\delta }=\left\{\left(x_{i},y_{j}\right):\left|P\left(DLT|\left(x_{i},y_{j}\right),z\right)-\theta \right|<\delta \right\}$ is the set of true MTDs such that the threshold parameter *δ* is fixed by a clinician. Following the same rationale, we also consider the percentage of selection of at least *K* dose combinations that are MTDs discussed in [35] is

(17) $${\text{PS}}_{K}=100\times \frac{1}{m}\sum_{i=1}^{m}I\left(\left|\Gamma_{i}\cap \Gamma_{\delta }\right|\geq K\right),$$

and the weighted average proportion of the recommended set of dose combinations which are MTDs is given by

(18) $${\text{S}}_{\Gamma_{\delta }}=\frac{\sum_{i=1}^{m}\left|\Gamma_{i}\cap \Gamma_{\delta }\right|}{\sum_{i=1}^{m}\left|\Gamma_{i}\right|}.$$

### 4.2. Illustration

We studied the two scenarios shown in Table 2 where each agent has five dose levels and target probability of DLT equal to *θ* = 0.33. The first scenario has low dose combinations as MTDs, while the second scenario has high dose combinations as MTDs. We simulated *m* = 3000 trials using the sample size of *n* = 42 patients and the same vague priors discussed in Section 2.

Table 3 shows the operating characteristics for safety and efficiency. The percentage of Grade 2, the average DLT rate and the percentage of excessive DLT are quite similar between models and criteria. On the other hand, the percentage that a prospective trial will recommend a set of dose combinations that are all MTDs (PS) and the percentage of selection of at least *K* dose combinations that are MTDs (S-K) favors ordinal models in comparison to the binary ones for both scenarios and models. Finally, the weighted average proportion of the recommended set of dose combinations which are MTDs (AV) have negligible differences between models. Similar to the continuous case discussed above, we note the superiority of CRM based designs relative to EWOC in recommending the MTD under Scenario 2.

## 5. Concluding Remarks

Clinical oncologists often advocate for a more comprehensive use of the CTCAE to characterize the toxicity profiles of cancer patients enrolled in clinical trials. Researchers have used various summary scores of toxicities to better ascertain patients’ adverse events burden to different cancer treatments with varying degree of success (see, e.g., [41] for the maximum-grade, [42] for the toxicity burden based on average and duration of low-grade toxicities, and [43,44] for the toxicity index). However, implementation of similar summary scores in dose finding early phase cancer trials is more challenging due to the sequential nature of these designs and the small sample size. In this manuscript, we extend the single agent trial design that accounts for lower grade toxicities [8] to drug combination trials using two different estimation criteria for dose allocation, EWOC and CRM. A proportional odds model for describing the relationship between dose combinations and the risk of ordinal toxicities was used and compared with models that use binary indicators of DLT. Extensive simulations under different practical scenarios for the location of the true MTD curve and true fraction of Grade 2 DLTs showed that, in most cases, the ordinal and binary models have similar safety profiles, regardless of the criteria used to estimate the next dose. We also observed that the ordinal model has a slightly higher pointwise percent selection relative to the binary model uniformly under half the scenarios and that, for each model, CRM outperforms EWOC with respect to pointwise percent recommendation uniformly across all scenarios. Therefore, the ordinal model using CRM criteria for dose estimation should be used to design prospective trials since this model results in fewer DLTs relative to the binary case when the MTD is far from the minimum dose combination, on the average, and it maintains its efficiency in estimating the MTD.

For single agent dose finding trials using EWOC with ordinal grade of toxicity, Tighiouart et al. [8] proved that if the maximum grade of toxicity experienced by patient (*k* − 1) is Grade 2, then the dose allocated to patient *k* is lower than the dose that would have been given to patient *k* had the maximum grade of toxicity experienced by patient (*k* − 1) been grade 0 or 1. This is an important property because it is not ethical to escalate the dose for the next patient by the same amount as the one had the current patient experienced a maximum of grade 0 or 1 toxicity. This property does not hold under model (2) and trial design described in Section 2.3. This is partly due to the lack of DLT attribution to either one or both drugs. In model (2), a DLT event is attributed to either drug *A*, drug *B*, or both and hence, dose escalation or de-escalation cannot be attributed to DLTs caused by either *A* or *B*. This is not an uncommon problem in cancer treatment since most DLTs are overlapping. However, a similar property was noted when the true MTD curve is far away from the minimum dose combination (Scenario 1), where a more cautious dose escalation towards the MTD was observed resulting in fewer patients exhibiting DLTs relative to binary models of DLT, on the average. For some class of drugs, clinicians are able to attribute certain toxicities to a particular drug under investigation. We plan to extend the work of Jimenez et al. [40] that models an unknown fraction of DLT attribution to account for lower dose toxicities and further explore this ethical property.

## Figures and Tables

**Table 1.** Average DLT rate and % trials: DLT rate > *θ* + 0.10 under Scenarios (1)–(6).

Scenario	Design	Average % Grade 2 (Z = 2)		Average % DLTs (Z = 3) (% Trials: DLT Rate (Z = 3) > 0.43)	

		Binary	Ordinal	Binary	Ordinal
1a	EWOC	76.12	81.41	16.31 (0.0)	10.86 (0.0)
	CRM	76.03	81.32	16.47 (0.0)	11.14 (0.0)
1b	EWOC	78.47	83.33	16.35 (0.0)	11.48 (0.0)
	CRM	78.76	84.34	16.16 (0.0)	10.48 (0.0)

2a	EWOC	61.22	61.77	30.31 (0.0)	29.37 (0.0)
	CRM	59.36	60.39	32.33 (0.23)	31.26 (0.47)
2b	EWOC	64.34	65.23	30.44 (0.07)	29.45 (0.40)
	CRM	62.32	67.80	32.45 (0.43)	31.67 (0.70)

3a	EWOC	67.65	69.63	25.29 (0.0)	22.96 (0.0)
	CRM	65.29	67.46	27.36 (0.0)	25.53 (0.0)
3b	EWOC	69.67	71.48	25.25 (0.0)	23.30 (0.0)
	CRM	67.40	69.27	27.53 (0.0)	25.64 (0.0)

4a	EWOC	58.98	58.47	32.64 (0.07)	33.06 (1.10)
	CRM	57.74	57.79	33.95 (0.20)	34.11 (1.57)
4b	EWOC	61.90	61.51	32.90 (0.03)	33.17 (0.97)
	CRM	60.73	60.37	34.06 (0.20)	34.45 (1.57)

5a	EWOC	49.11	48.44	36.73 (2.63)	37.66 (6.30)
	CRM	48.38	47.73	37.00 (2.17)	38.55 (10.20)
5b	EWOC	57.39	55.96	36.71 (2.33)	38.14 (8.97)
	CRM	57.06	55.10	36.96 (2.13)	38.98 (13.40)

6a	EWOC	52.04	52.19	32.83 (1.00)	32.67 (1.47)
	CRM	50.45	50.98	34.98 (2.77)	34.65 (3.50)
6b	EWOC	61.04	61.17	32.85 (1.20)	32.70 (2.00)
	CRM	59.11	59.32	34.91 (2.20)	34.63 (4.40)

**Table 2.** A selected dose limiting toxicity scenario with *θ* = 0.33 for *Z* = 1, 2 considering discrete dose combinations. True MTDs are shown in bold.

Scenario 01										

Dose Level	Z = 1					Z = 2				

	1	2	3	4	5	1	2	3	4	5
5	0.50	0.44	0.38	0.31	0.24	0.45	0.53	0.60	0.68	0.75
4	0.53	0.49	0.43	0.35	0.26	0.40	0.46	0.53	0.63	0.70
3	0.58	0.57	0.51	0.43	0.36	**0.33**	0.36	0.44	0.53	0.59
2	0.65	0.57	0.55	0.47	0.44	0.20	**0.33**	0.38	0.48	0.53
1	0.68	0.65	0.58	0.53	0.48	0.15	0.20	**0.33**	0.40	0.47

Scenario 02										

Dose Level	Z = 1					Z = 2				

	1	2	3	4	5	1	2	3	4	5
5	0.36	0.44	0.48	0.41	0.34	0.28	0.35	0.42	0.52	0.60
4	0.30	0.39	0.43	0.45	0.38	0.22	0.23	**0.33**	0.43	0.45
3	0.24	0.27	0.31	0.43	0.49	0.17	0.20	0.21	**0.33**	0.39
2	0.15	0.27	0.28	0.37	0.44	0.11	0.14	0.19	0.25	0.30
1	0.10	0.25	0.28	0.30	0.38	0.08	0.13	0.15	0.21	0.27

**Table 3.** Operating characteristics summarizing trial efficiency and safety for CRM and EWOC using non-informative priors.

Scenario 01								

Criterion	Model	PS	S-3	S-2	S-1	AV	% Grade 2	Average DLT Rate (% Excessive DLT)
EWOC	Binary	57.2	9.5	29.0	64.6	84.8	54.33	31.91 (1.27)
	Ordinal	71.7	26.7	54.7	92.2	82.4	54.81	31.59 (1.63)

CRM	Binary	54.2	8.2	25.3	62.7	82.4	53.74	32.92 (1.66)
	Ordinal	70.5	26.2	61.9	91.6	81.1	53.86	32.87 (2.43)

Scenario 02								

Criterion	Model	PS	S-3	S-2	S-1	AV	% Grade 2	Average DLT Rate (% Excessive DLT)
EWOC	Binary	26.6	3.3	12.5	40.1	64.2	30.14	21.11 (0.00)
	Ordinal	38.4	38.2	58.4	84.9	63.9	30.95	21.30 (0.00)

CRM	Binary	29.2	3.7	12.6	40.0	68.8	31.57	23.24 (0.04)
	Ordinal	44.7	49.9	69.4	90.5	71.3	32.77	23.33 (0.00)
